# Impact of Intraoperative Neurophysiological Monitoring on Supratentorial Meningioma Surgery: A Systematic Review

**DOI:** 10.3390/diagnostics16172730

**Published:** 2026-08-26

**Authors:** Martina Offi, Renata Martinelli, Benedetta Burattini, Michele Di Domenico, Francesca Annunziata, Francesco Pambianco, Rosa Linda Rinaldi, Vedant Vikram, Alessandro Izzo, Manuela D’Ercole, Rina Di Bonaventura, Alessandro Olivi, Francesco Doglietto, Quintino Giorgio D’Alessandris, Nicola Montano

**Affiliations:** 1 Department of Neuroscience, Section of Neurosurgery, Università Cattolica del Sacro Cuore, Largo F. Vito, 1, 00168 Roma, Italy; martina.offi@guest.policlinicogemelli.it (M.O.); renata.martinelli@guest.policlinicogemelli.it (R.M.); benedetta.burattini@guest.policlinicogemelli.it (B.B.); francesca.annunziata@guest.policlinicogemelli.it (F.A.); francesco.pambianco@guest.policlinicogemelli.it (F.P.); rosalinda.rinaldi01@icatt.it (R.L.R.); vedant.vikram01@icatt.it (V.V.); alessandro.izzo@policlinicogemelli.it (A.I.); rina.dibonaventura@policlinicogemelli.it (R.D.B.); alessandro.olivi@policlinicogemelli.it (A.O.); francesco.doglietto@policlinicogemelli.it (F.D.); quintinogiorgio.dalessandris@policlinicogemelli.it (Q.G.D.); 2 Department of Neurosurgery, Fondazione Policlinico Universitario A. Gemelli IRCCS, Largo A. Gemelli, 8, 00168 Roma, Italy; michele.didomenico@policlinicogemelli.it (M.D.D.); manuela.dercole@policlinicogemelli.it (M.D.)

**Keywords:** brain mapping, intraoperative monitoring, meningioma, neurological outcomes, supratentorial neoplasms, systematic review

## Abstract

**Objectives:** Our objective was to systematically evaluate the role of intraoperative neurophysiological monitoring (IONM) in supratentorial meningioma surgery, focusing on intraoperative changes, surgical decision-making, and postoperative neurological outcomes. **Methods**: A PRISMA-guided systematic review was conducted across PubMed, Scopus, and Cochrane databases. Nine studies, including 576 supratentorial meningioma (STM) cases identified across the source cohorts, were analyzed. Data extraction included study design, tumor location, monitoring modalities, intraoperative neurophysiological changes, and postoperative outcomes. Risk of bias was assessed using ROBINS-I, the JBI Critical Appraisal Checklist, or QUADAS-2, according to study design. **Results**: IONM modalities varied widely and included motor and somatosensory evoked potentials, cortical stimulation, and electromyography. Substantial heterogeneity was observed. Postoperative deficits occurred in both monitored and unmonitored patients (5.2–66%), including cases with preserved intraoperative signals, suggesting false negatives. IONM influenced intraoperative decision-making in selected cases, particularly for tumors near motor areas. However, its impact on postoperative outcomes remains unclear. **Conclusions**: Intraoperative motor cortex identification was the most consistently reported finding with a surgical impact. Available data from the literature are inconclusive about the role of IONM in reducing postoperative neurological morbidity.

## 1. Introduction

Meningiomas are the most common primary intracranial tumors in adults, accounting for over one-third of all central nervous system neoplasms [1]. They are typically benign, extra-axial tumors with a clear dissection plane, often allowing for complete surgical resection. However, the surgical challenge significantly increases when meningiomas are located near or within eloquent brain regions (such as the primary motor cortex, somatosensory cortex, or visual pathways). In these cases, tumor adherence to critical neurovascular structures and loss of the arachnoid plane may lead to a risk of postoperative neurological deficits [2,3].

Intraoperative neurophysiological monitoring (IONM) has become an established tool in various neurosurgical procedures, where it supports functional mapping and real-time identification of neurological risks during resection [4,5,6,7,8]. Its application has been shown to reduce neurological morbidity and guide surgical decisions, especially in surgeries involving eloquent cortical or subcortical areas [9,10,11]. In this context, functional mapping and continuous neurophysiological monitoring should be distinguished: mapping is primarily used to localize eloquent neural structures, whereas monitoring aims to detect functional changes during surgical manipulation.

Currently, IONM has an established role in neurosurgery for intra-axial tumors and for posterior fossa lesions, including meningiomas, especially those located in the cerebellopontine angle [4,5,6,7,8]. However, notwithstanding some pioneering studies [12,13], its implementation in the surgical treatment of supratentorial meningiomas remains inconsistent. Therefore, no standardized protocols or recommendations exist to guide its application in this setting.

The objective of this systematic review was to gather and critically analyze the available literature on the use of IONM during neurosurgery for the resection of supratentorial meningiomas. We focused on the impact of IONM on surgical decision-making and postoperative neurological outcomes.

## 2. Materials and Methods

**Figure 1:**
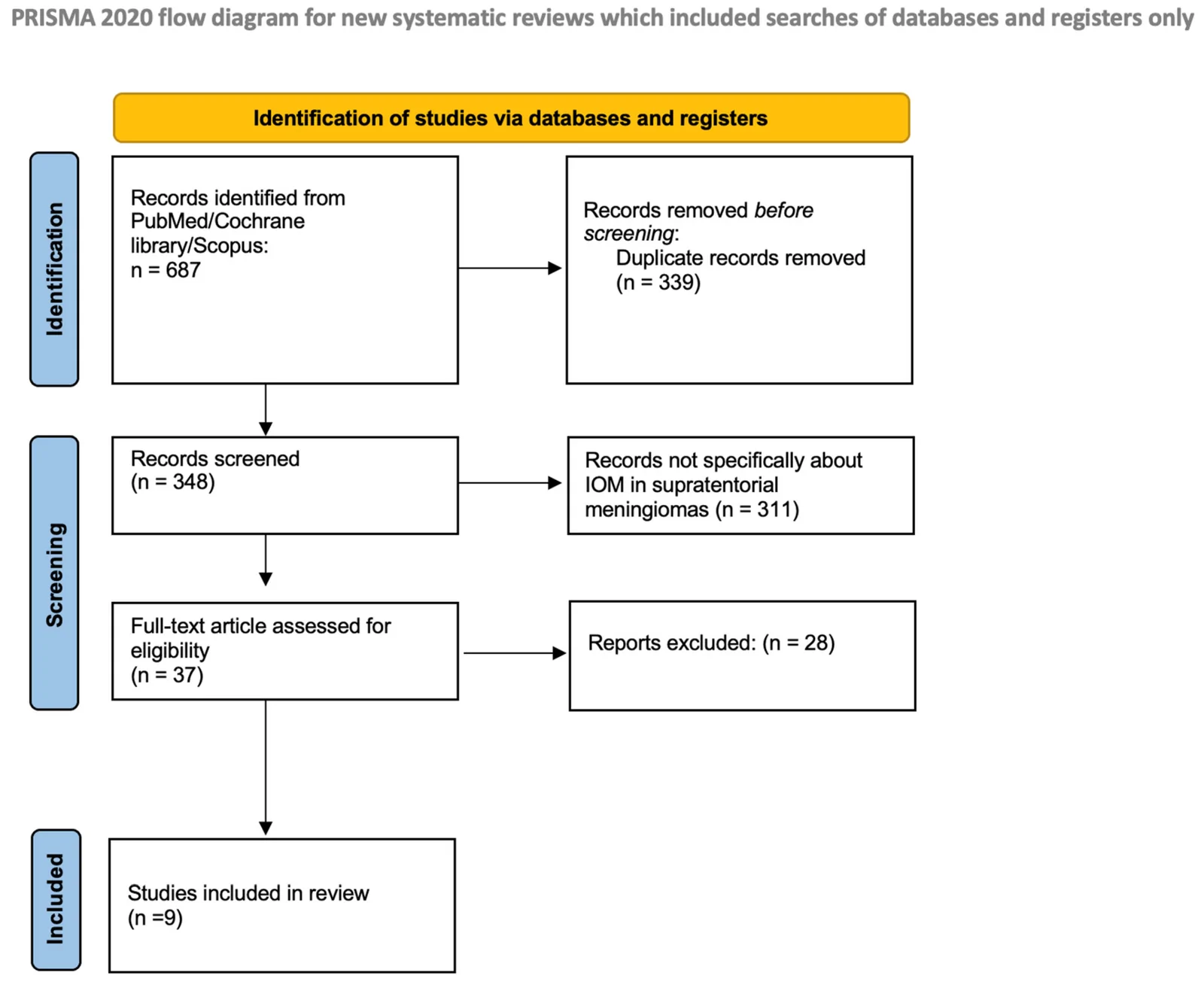
PRISMA 2020 flow diagram illustrating the selection process of studies included in the review.

This systematic review was conducted in accordance with the Preferred Reporting Items for Systematic Reviews and Meta-Analyses (PRISMA) guidelines to ensure transparency and methodological rigor. The PRISMA flowchart (Figure 1) is included to illustrate the selection process [14]. The review protocol adhered to the recommendations of the Cochrane Handbook for Systematic Reviews of Interventions.

Three databases (PubMed, Scopus, and Cochrane) were screened for relevant studies using the following search terms: (“Intraoperative monitoring” OR “evoked potentials” OR “neurophysiological monitoring” OR “electrophysiological monitoring”) AND (“supratentorial” OR “cranial” OR “cerebral” OR “CNS”) AND (“meningioma” OR “extra-axial tumor”); the complete search string (database-specific syntax and date limits) is provided in full in the Supplementary Materials File S1. Only reports written in English were considered. Case reports mentioning the use of IONM in supratentorial meningiomas were excluded. Reports involving tentorial, posterior fossa, spinal or extracranial meningiomas were excluded. The final search was performed on 15 November 2025.

Three independent reviewers (M.O., R.M., B.B.) screened the abstracts and full-text articles. Discrepancies between reviewers were resolved by a senior reviewer (N.M.). Data extraction was independently performed by two reviewers, and any discrepancies were resolved by consensus. Data extracted included demographic information, radiological assessments, clinical history, treatment details, indications, surgical approach, type of intraoperative monitoring used, intraoperative modifications of neurophysiological parameters, postoperative clinical outcomes, and overall surgical outcomes. Additionally, surgical techniques for meningioma resection and the impact of intraoperative monitoring on the surgical workflow were evaluated. Risk of bias was assessed using three tools selected by study design: ROBINS-I for the two comparative studies with an internal non-monitored group (Supplementary Materials Figure S1); the JBI Critical Appraisal Checklist for Case Series for the five single-arm series (Supplementary Materials Table S2) and QUADAS-2 for the two diagnostic accuracy studies (Supplementary Materials Figure S2).

This systematic review was registered in PROSPERO, with number CRD420251245568.

## 3. Results

A total of 687 records were identified through database searching (PubMed *n* = 211; Scopus *n* = 470; Cochrane Library *n* = 6).

Duplicates were removed using Covidence software (*n* = 326 automatically identified). An additional 13 duplicates were manually identified and excluded after reviewer verification. A total of 348 unique records remained for title and abstract screening.

Thirty-seven studies were selected for full-text assessment. Of the 28 excluded studies, eight were excluded due to a non-supratentorial meningioma population (P), 14 due to the absence of detailed neurophysiological intraoperative monitoring as the intervention of interest (I), and six due to ineligible study design (five case reports and one review) according to the predefined study design criteria (S) of the PICOS framework; no exclusions were attributable to comparison or outcome (O) criteria (Supplementary Table S1). Nine articles met the predefined eligibility criteria and were included in the final qualitative analysis (Figure 1), accounting for a cumulative number of 576 supratentorial meningioma (STM) cases identified across the source cohorts (Table 1). All included studies were retrospective observational series. In the series from Policicchio et al. [15], only one meningioma case was described. Two studies included cases operated on both using and not using IONM [16,17]; however, only the study by Shin et al. [17] adopted a formal case–control design.

Patient age ranged from 34 to 60 years, with slight variability among cohorts.

The clinical features of the included studies and patients are summarized in Table 2. The most common meningioma locations were convexity meningiomas, particularly close to the motor area; parasagittal/falcine meningiomas; and anterior and middle skull base. Tumor grading was reported in a minority of studies, while the extent of resection was most consistently described.

IONM techniques and study results are reported in Table 3 and Table 4. When supratentorial meningioma-specific outcomes were not separately extractable, results from the whole source cohort are explicitly identified and should not be interpreted as STM-specific estimates. Various IONM modalities were employed, namely somatosensory evoked potentials (SSEPs), motor evoked potentials (MEPs), cortical mapping (CM), visual evoked potentials (VEPs), triggered electromyography (t-EMG), free-running electromyography (f-EMG), electroretinogram (ERG) and train of four (TOF). MEPs and SSEPs were the most consistently used techniques, described in all but two included studies.

Some studies reported an intraoperative modification in surgical strategy in response to IONM alarms, ranging from adjustments in resection margins to the complete abortion of the surgical procedure [12,18,19,20,21,22].

### Risk of Bias Assessment

Risk of bias was assessed using three tools selected by study design: ROBINS-I for the two comparative studies [16,17], the JBI Critical Appraisal Checklist for the five single-arm case series [12,15,18,19,20], and QUADAS-2 for the two diagnostic/prognostic accuracy studies [21,22]. Both studies assessed using ROBINS-I were judged to be at serious overall risk, mainly due to confounding by indication. Among the case series, four were low-risk and one was moderate. Of the QUADAS-2 studies, Kim showed some concerns and Jimenez was high-risk, reflecting its case–control design. This heterogeneity across tools confirms the scarcity of high-quality evidence on IONM in supratentorial meningioma surgery. Full domain-level judgements, traffic-light plots, and the JBI summary table are provided in the Supplementary Materials.

## 4. Discussion

The use of IONM during neurosurgical procedures is a “hot topic” in the scientific literature. A search of the PubMed database using the string “IONM neurosurgery” yielded 303 results published in the last 5 years (2021–2025), compared to less than 200 items published between 2005 and 2020. In this scenario, the lack of data regarding IONM use in supratentorial meningiomas, which are one of the most common neurosurgical diseases, is somewhat surprising. The inconsistent availability of an IONM service in neurosurgical centers, and a perceived low risk of postoperative deficits in some cases, could explain this finding. In the present study, we aimed to provide an up-to-date overview of the existing scientific literature, with the aim of fostering future studies.

Among the selected manuscripts, only Shin et al. formally compared outcomes between patients undergoing surgery with and without IONM [17], comparing a series of 99 meningioma patients in whom IONM was used vs. 107 patients from a historical cohort in which IONM was not used. Different locations were included; supratentorial meningiomas accounted for 85 of the 99 monitored patients and 93 of the 107 historical patients. Unfortunately, specific data for supratentorial cases were not reported. In the overall patient cohort, IONM triggered alarm signals in 10 patients, leading to the termination of surgery in two of them. Postoperative sequelae were noted in 7 of these 10 patients (70%) vs. 5 of the 89 patients without IONM warnings (5.6%); thus, the total rate of postoperative morbidity in monitored cases was 12/99 (12.1%). In the unmonitored cohort, 12 of 107 patients (11.2%) experienced postoperative morbidity (*p* > 0.99, Fisher’s exact test). Thus, the authors concluded that, while IONM guided intraoperative decisions, it did not reduce overall morbidity. The other study in which both IONM-monitored and unmonitored cases were included was Eichberg et al. [16]; however, the focus of the paper was the optimization of surgical strategy in parasagittal or falcine meningiomas, and not specifically IONM. MEPs were employed in 19 out of 58 patients (32.8%). The authors note that changes in MEPs could lead to an early termination of tumor resection to prevent neurological injury, thus indicating that IONM may have influenced surgical strategy in selected cases. However, specific neurophysiological changes and corresponding surgical modifications were not consistently documented.

Paldor et al. analyzed the impact of IONM in the surgical workflow on 40 patients undergoing surgery for supratentorial meningiomas [20]. They suggested a classification system to categorize IONM-induced workflow changes, ranging from Class 1 (premature surgery stop) to Class 5 (no changes). In 60% of cases, IONM influenced the surgical workflow (Classes 1–4), with the most common event being a change in resection strategy after motor cortex localization. In these cases, postoperative outcomes were more favorable compared to cases in which IONM did not influence surgical workflow (Class 5). In fact, the percentages of transient and permanent deficits were 8.3% and 0% for Classes 1–4 vs. 18.75% and 6.25% for Class 5, respectively. A limitation of the study is the lack of details on Class 5 cases; in particular, it is not disclosed whether the continuation of the original surgical strategy was explained by the absence of an IONM alarm or by a surgeon’s decision. Another weak point of the study is the absence of a control group without IONM.

Tumors located in proximity to the motor strip were the focus of three papers. Ebeling et al. [12] reported on 50 patients, 15 of whom harbored meningiomas, finding that intraoperative identification of the precentral gyrus led to a change in surgical strategy in 14% of cases. As a result, only a small number of patients (2 out of 50) experienced mild postoperative hemiparesis. However, results are reported for the entire study cohort, and no meningioma-specific data were available. Ostry et al. [19] retrospectively analyzed 42 rolandic meningiomas resected using motor cortex mapping, MEPs and SSEPs. The primary motor cortex was intraoperatively identified in all patients through direct cortical mapping, and this led to deliberate subtotal resection in cases in which the dissection plane on the motor cortex was lost. Finally, Policicchio et al. [15] studied 25 patients with brain lesions adjacent to motor areas to evaluate the role of IONM and other tools to aid surgical resection; one patient harbored an anaplastic meningioma. In this case, the primary motor area was successfully identified intraoperatively, with no new postoperative neurological deficits.

Skull base meningiomas were described in four studies. Son et al. [18] presented a case series of 11 patients undergoing surgery for middle fossa tumors using a transzygomatic approach, including three sphenoidal meningiomas. All patients were monitored using MEP and free-run EMG. In meningioma cases, IONM showed limited sensitivity, with two of three patients developing third nerve palsy, without intraoperative warning signs. Jimenez et al. [21] analyzed a cohort of patients who underwent endoscopic skull base surgery, including six meningioma cases, in whom the abducens nerve was stimulated using t-EMG. A case–control design was adopted with the aim of comparing IONM data between patients with a new postoperative abducens nerve palsy (cases) and those without palsy (controls). A number of parameters (presence of f-EMG activity, lack of evoked compound muscle action potential (CMAP) response to stimulation, decreased CMAP amplitude, and increased CMAP onset latency) were associated with acute postoperative abducens nerve palsy, while long-term palsy was associated with t-EMG data. No specific data regarding meningioma cases were provided.

A recent large single-center retrospective series by Kim et al. [22] specifically investigated the diagnostic performance of intraoperative transcranial MEPs during intracranial meningioma surgery. Among the 319 enrolled patients, 233 were affected by supratentorial meningiomas. Intraoperative TES-MEP changes were detected in 5% of the overall cohort, while immediate postoperative motor deficits occurred in 3.8%. TES-MEP monitoring demonstrated a high overall diagnostic accuracy (96.9%) and specificity (97.7%) for predicting early postoperative motor deficits. However, sensitivity was reduced in falcine meningiomas, where false-negative results were more frequent, highlighting the anatomical limitations of TES-MEP in midline and parasagittal locations. Importantly, this study primarily assessed the diagnostic accuracy of TES-MEPs for predicting postoperative motor deficits rather than the clinical efficacy of IONM in reducing neurological morbidity; its findings should therefore be interpreted separately from comparative effectiveness data.

Although not included in the present systematic review due to their case report design, several illustrative cases in the literature highlight the potential role of IONM in supratentorial meningioma surgery. Silverstein et al. [23] reported a case of intraoperative vascular injury during resection of a planum sphenoidale meningioma, in which the sudden loss of ipsilateral lower-limb MEPs enabled the surgical team to promptly recognize and repair an unrecognized injury to the contralateral anterior cerebral artery complex. Postoperatively, the patient developed transient lower-limb weakness, with complete recovery at three months. Mori et al. [24] described the case of a pregnant woman presenting with visual field deficits who underwent resection of a suprasellar meningioma compressing the optic nerve and chiasm; intraoperative improvement in VEPs accurately predicted postoperative visual recovery. Finally, Silverstein et al. [25] reported an unusual intraoperative decline in SSEPs during meningioma surgery related to patient positioning, which allowed for the prompt correction and prevention of permanent neurological injury.

The wide variability in reported postoperative neurological deficits across studies (approximately 5–66%) should not be interpreted as a pooled STM-specific estimate because some percentages derive from mixed cohorts and the studies differ substantially in tumor location, neurological outcomes, follow-up, and monitoring strategies.

Studies reporting the highest rates of postoperative deficits predominantly included skull base meningiomas, in which morbidity is mainly related to cranial nerve dysfunction, an outcome that standard MEP and SSEP monitoring may fail to detect when specific cranial nerve-targeted techniques are not systematically applied [18,21]. In contrast, studies focusing on convexity, parasagittal, or falcine meningiomas, where the primary surgical risk involves the motor cortex or corticospinal tracts, generally reported lower rates of postoperative deficits, particularly when multimodal IONM combining cortical mapping, MEPs, and SSEPs was employed [16,19,20]. These factors may help explain the divergent conclusions of Shin et al. and Paldor et al. [17,20]. Shin et al., analyzing a large and anatomically heterogeneous cohort using MEPs and SSEPs, found that IONM influenced intraoperative decision-making but did not reduce overall postoperative morbidity. Conversely, Paldor et al. focused on tumors adjacent to motor areas and emphasized workflow modifications driven by cortical mapping, a setting in which IONM may influence surgical behavior, although the absence of a non-IONM control group limits conclusions regarding clinical efficacy.

### Limitations

The limited amount of available data, the scarcity of comparative studies, and the risk of bias of the included studies result in the overall quality of the available evidence being considered low to moderate, thus warranting further studies on the topic. Furthermore, the scarcity of comparative studies and inconsistency of reported outcomes prevented us from performing a meta-analysis. Moreover, the predominantly retrospective, single-center nature of the available evidence, together with heterogeneity in tumor location, monitoring protocols, and outcome definitions, limits the external validity and generalizability of the findings. Finally, the restriction to English-language publications may have introduced language bias and should be considered when interpreting the generalizability of our findings.

## 5. Conclusions

We presented the current evidence in the literature on the use of IONM during surgery for supratentorial meningiomas.

The only comparative study with sufficient statistical power failed to demonstrate any clinical advantage of IONM over standard surgery. While IONM may provide useful real-time intraoperative information and influence surgical strategy in selected cases, the available evidence is insufficient to determine whether this intraoperative utility translates into improved postoperative neurological outcomes. Intraoperative motor cortex identification was the most consistently reported finding with a surgical impact. Prospective comparative studies are needed specifically for rolandic meningiomas, where preliminary evidence from three case series suggests potential benefit, but adequately controlled data remain absent.

## Figures and Tables

**Table 1:** Summary of included studies.

Author, Year	Type	*n* STMs
Ebeling, 1992 [12]	Retrospective	15
Son, 2012 [18]	Retrospective	3
Ostry, 2012 [19]	Retrospective	42
Eichberg, 2020 [16]	Retrospective	58
Policicchio, 2021 [15]	Retrospective	1
Paldor, 2022 [20]	Retrospective	40
Shin, 2024 [17]	Retrospective	178
Jimenez, 2024 [21]	Retrospective	6
Kim, 2025 [22]	Retrospective	233

**Table 2:** Clinical features of supratentorial meningiomas described in the included studies.

Author, Year	*n* pts	*n* STMs	Localization (*n*)	Histology (WHO Grade) (*n*)	EOR (*n*)
Ebeling, 1992 [12]	50	15	N/A	N/A	N/A
Son, 2012 [18]	11	3	Skull base (3)	N/A	GTR (3)
Ostry, 2012 [19]	42	42	Convexity (25) Parasagittal (10) Falx (7)	1 (28), 2 (14)	GTR (30), STR (12)
Eichberg, 2020 [16]	58	58	Parasagittal (45) Falx (13)	N/A	GTR (30), STR (28)
Policicchio, 2021 [15]	25	1	Convexity (1)	G3 (1)	GTR (1)
Paldor, 2022 [20]	40	40	Parasagittal (22) Convexity (16) Falx (2)	G1 (37), G2 (3)	GTR (32), STR (5), PR (1)
Shin, 2024 [17]	206	178	Convexity (57) Parasagittal (48) Falx (11) Skull base (57) Intraosseous (2) Ventricle (3)	N/A	N/A
Jimenez, 2024 [21]	56	6	Skull base (6)	N/A	N/A
Kim, 2025 [22]	319	233	Convexity (91) Parasagittal (56) Falx (24) Skull base (88) Ventricle (5)	N/A	N/A

EOR, extent of resection; GTR, gross total resection; N/A, not available; PR, partial resection; pts, patients; STMs, supratentorial meningiomas; STR, subtotal resection.

**Table 3:** Studies including monitored and non-monitored cases of supratentorial meningioma surgery.

Author, Year	*n* STMs	*n* IONM Cases	IONM Type	I/O IONM Changes	Postoperative Deficits	Conclusions
Eichberg, 2020 [16]	58	19	MEPs	N/A	3/58 (5%)	IONM impact on surgical strategy; one patient with intact MEP had deficit, suggesting false negative
Shin, 2024 [17]	178	85	MEPs, SSEPs	10.1% *^§^	* IONM: 12.1% 70% IONM change 5.6% no IONM change * no IONM: 11.2%	IONM guided intraoperative decisions but did not reduce overall morbidity

I/O, intraoperative; IONM, intraoperative neuromonitoring; MEPs, motor evoked potentials; SSEPs, somatosensory evoked potentials; STMs, supratentorial meningiomas. * Percentages are calculated on the whole series including infratentorial meningiomas (99 IONM, 107 no IONM). ^§^, of IONM cases.

**Table 4:** Details on noncomparative studies.

Author, Year	STMs	IONM Type	I/O IONM Changes	Postoperative Deficits	FU (mos)	Conclusions
Ebeling, 1992 [12]	15	CM, MEPs, SSEPs	NA	4% *	6	IONM led to a revision of surgical strategy in tumors located close to the motor area
Son, 2012 [18]	3	MEP, f-EMG	0	66.7%	N/A	Postoperative 3rd nerve palsy without IONM warnings
Ostry, 2012 [19]	42	CM, MEPs, SSEPs,	N/A	11.9% (temporary) 7.1% (permanent)	33.2	Cortical mapping identified the motor area in all cases; IONM signal changes were not predictive of postoperative deficits
Policicchio, 2021 [15]	1	MEPs, SSEPs	N/A	0	6	Successful resection of a complex rolandic meningioma
Paldor, 2022 [20]	40	CM, MEP, SSEP	60%	IONM change: 8.3% temporary, 0 permanent No IONM change: 18.8% temporary, 6.3% permanent	16	Elaboration of a score describing IONM impact on surgical strategy with prognostic impact
Jimenez et al., 2024 [21]	6	t-EMG and f-EMG	N/A	67% *	N/A	Analysis of EMG parameters predicted postoperative abducens nerve palsy
Kim, 2025 [22]	233	MEPs, SSEPs	5% *	3.8% *	N/A	Intraoperative TES-MEP demonstrated a high diagnostic accuracy and high specificity in predicting immediate postoperative motor deficits

CM, cortical mapping; EMG, electromyography; f-EMG, free-run EMG; t-EMG, triggered EMG; I/O, intraoperative; IONM, intraoperative neuromonitoring; MEPs, motor evoked potentials; SSEPs, somatosensory evoked potentials; STMs, supratentorial meningiomas. * Percents are calculated on the whole series including non-STM patients.

## Data Availability

No new data were created or analyzed in this study. Data sharing is not applicable to this article.

## Supplementary Materials

The following supporting information can be downloaded at: https://www.mdpi.com/article/10.3390/diagnostics16172730/s1, Supplementary Materials File S1: Detailed search strategy; Table S1: Full-text excluded studies. Table S2. JBI Critical Appraisal Checklist for Case Series. Figure S1. Robins-I tool for comparative retrospective cohorts. Figure S2. QUADAS 2 tool for diagnostic/predictive studies.

## Abbreviations

The following abbreviations are used in this manuscript:

CMAP	Compound Muscle Action Potential
CM	Cortical Mapping
CNS	Central Nervous System
ERG	Electroretinogram
f-EMG	Free-running Electromyography
IONM	Intraoperative Neurophysiological Monitoring
MEP	Motor Evoked Potential
PICOS	Population, Intervention, Comparison, Outcomes, Study Design
PRISMA	Preferred Reporting Items for Systematic Reviews and Meta-Analyses
PROSPERO	International Prospective Register of Systematic Reviews
ROBINS-I	Risk of Bias in Non-randomized Studies of Interventions
SSEP	Somatosensory Evoked Potential
TES-MEP	Transcranial Electrical Stimulation MEP
TOF	Train of Four
t-EMG	Triggered Electromyography
VEP	Visual Evoked Potential
