# Supplementary material for: Impact of Intraoperative Neurophysiological Monitoring on Supratentorial Meningioma Surgery: A Systematic Review
Source: Diagnostics (Basel). 2026 Aug 26;16(17):2730. doi: 10.3390/diagnostics16172730 (PMC13565359; doi:10.3390/diagnostics16172730)
Supplement: Supplementary file 1 [file diagnostics-16-02730-s001.zip › Supplementary Material S1.pdf]

**Supplementary Material S1 – Search Strategy and Study Selection**

# Supplementary Material S1

## Detailed Search Strategy

### Search Date

15 November 2025

## Database 1: PubMed

**Coverage:** 1963 – 15 November 2025
**Filters applied:** None
**Total records retrieved:** 211

### Search strategy:

```
(
  ("Intraoperative Monitoring"[Mesh]
   OR "Evoked Potentials"[Mesh]
   OR intraoperative monitoring[Title/Abstract]
   OR intra-operative monitoring[Title/Abstract]
   OR neurophysiological monitoring[Title/Abstract]
   OR electrophysiological monitoring[Title/Abstract]
   OR intraoperative neurophysiology[Title/Abstract]
   OR IONM[Title/Abstract]
   OR motor evoked potentials[Title/Abstract]
   OR somatosensory evoked potentials[Title/Abstract]
   OR MEP[Title/Abstract]
   OR SSEP[Title/Abstract])
)
AND
(
   supratentorial[Title/Abstract]
   OR cranial[Title/Abstract]
   OR cerebral[Title/Abstract]
   OR intracranial[Title/Abstract]
   OR brain[Title/Abstract]
   OR "Central Nervous System"[Mesh]
)
AND
(
   "Meningioma"[Mesh]
   OR meningioma*[Title/Abstract]
   OR extra-axial tumor*[Title/Abstract]
   OR extraaxial tumor*[Title/Abstract]
)
```

## Database 2: Scopus

**Coverage:** 1971 – November 2025
**Filters applied:** None
**Total records retrieved:** 470

### Search strategy:

```
TITLE-ABS-KEY(
(
  "intraoperative monitoring"
  OR "intra-operative monitoring"
  OR "neurophysiological monitoring"
  OR "electrophysiological monitoring"
  OR "intraoperative neurophysiology"
  OR "evoked potentials"
  OR "motor evoked potentials"
  OR "somatosensory evoked potentials"
  OR IONM
  OR MEP
  OR SSEP
)
AND
(
  supratentorial
  OR cranial
  OR cerebral
  OR intracranial
  OR brain
  OR "central nervous system"
)
AND
(
  meningioma*
  OR "extra-axial tumor"
  OR "extraaxial tumor"
  OR "extra-axial tumors"
  OR "extraaxial tumors"
)
)
```

## Database 3: Cochrane Library

**Coverage:** 1971 – November 2025
**Filters applied:** None
**Total records retrieved:** 6

### Search strategy:

(intraoperative monitoring OR neurophysiological monitoring OR IONM OR MEP OR SSEP OR "evoked potentials")

AND

(meningioma OR meningiomas)

## Study Selection Process

A total of 687 records were identified through database searching (PubMed n = 211; Scopus n = 470; Cochrane Library n = 6).

Duplicates were removed using Covidence software (n = 326 automatically identified). An additional 13 duplicates were manually identified and excluded after reviewer verification. A total of 348 unique records remained for title and abstract screening.

Thirty-seven studies were selected for full-text assessment. Of these, nine met the predefined eligibility criteria and were included in the final qualitative analysis (Figure 1). Twenty-eight full-text articles were excluded, and the specific reasons for exclusion are detailed in Supplementary Table S1.
