# Supplementary material for: Impact of Intraoperative Neurophysiological Monitoring on Supratentorial Meningioma Surgery: A Systematic Review
Source: Diagnostics (Basel). 2026 Aug 26;16(17):2730. doi: 10.3390/diagnostics16172730 (PMC13565359; doi:10.3390/diagnostics16172730)
Supplement: Supplementary file 1 [file diagnostics-16-02730-s001.zip › Supplementary Tables and Figures.pdf]

Supplementary Table S1

Full-text excluded studies

	Author	*Year*	*PMID*	*Reason for Exclusion*
1	Schramm	1988	3259682	Not supratentorial meningiomas
2	Couillard	1996	8607066	Case report
3	Schmid-Elsaesser	1997	9361054	Not detailed neurophysiological IONM
4	Black	1999	10493363	Not detailed neurophysiological IONM
5	Romstöck	2000	11014536	Not supratentorial meningiomas
6	Gorbach	2004	15599965	Not detailed neurophysiological IONM
7	Martorano	2007	17185033	Not detailed neurophysiological IONM
8	Goto	2010	21772800	Not supratentorial meningiomas
9	Kasper	2010	21245945	Not detailed neurophysiological IONM
10	Ferroli	2011	20960352	Not detailed neurophysiological IONM
11	Li	2011	21530073	Not detailed neurophysiological IONM
12	Matthies	2011	21798660	Not supratentorial meningiomas
13	Yang	2012	23372996	Not supratentorial meningiomas
14	Muroi	2013	23247024	Not supratentorial meningiomas
15	Della Puppa	2014	24484251	Not detailed neurophysiological IONM
16	Prada	2015	26193670	Not detailed neurophysiological IONM
17	Lekht	2016	26926064	Not detailed neurophysiological IONM
18	Chakrabarti	2017	28235530	Not detailed neurophysiological IONM
19	Acerbi	2018	29852759	Not detailed neurophysiological IONM
20	Güngör	2019	31121376	Not detailed neurophysiological IONM
21	Bailo	2019	31560106	Review
22	Lepski	2021	32671694	Not supratentorial meningiomas
23	Ibn Essayed	2021	34498699	Not detailed neurophysiological IONM
24	D'Ercole	2023	37511716	Not supratentorial meningiomas
25	Silverstein	2023	—	Case report (n=1)
26	Silverstein	2024	39163123	Case report (n=1)
27	Mori	2024	39455945	Case report (n=1)
28	Dadario	2021	34781833	Case report (n=1)

Supplementary Material Figure S1. Robins-I tool for comparative retrospective cohorts.

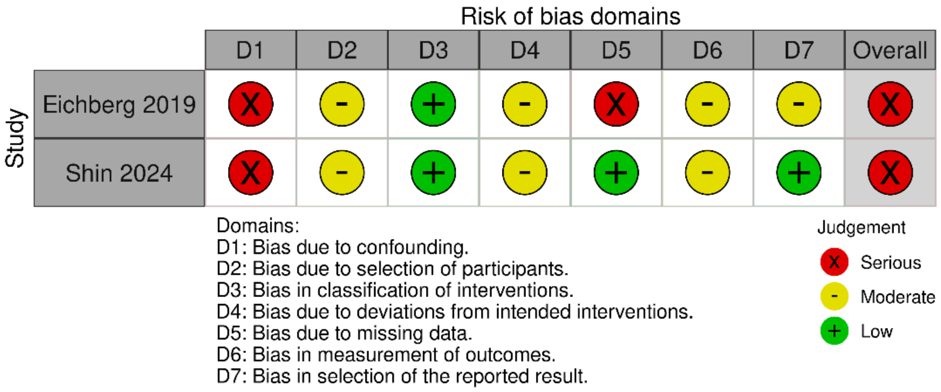

Supplementary Material Figure S2. QUADAS 2 tool for diagnostic/predictive studies.

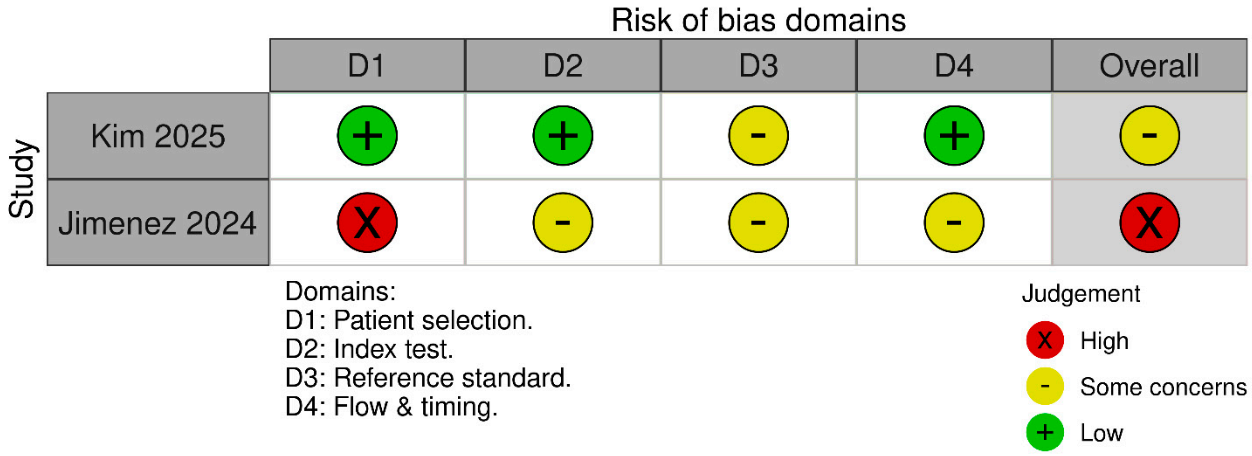

Supplementary Material Table S2. JBI Critical Appraisal Checklist for Case Series.

Study	Q1	Q2	Q3	Q4	Q5	Q6	Q7	Q8	Q9	Q10	Overall
Ebeling 1992	Y	Y	Y	Y	Y	Y	Y	Y	Y	Y	Low
Son 2012	Y	Y	Y	U	U	Y	Y	U	Y	Y	Moderate
Ostry 2012	Y	Y	Y	Y	Y	Y	Y	Y	Y	Y	Low
Policicchio 2021	Y	Y	Y	Y	Y	Y	Y	Y	Y	Y	Low
Paldor 2022	Y	Y	Y	Y	Y	Y	Y	Y	Y	Y	Low

References

1. Schramm J, Mokrusch T, Fahlbusch R, Hochstetter A. Detailed analysis of intraoperative changes monitoring brain stem acoustic evoked potentials. *Neurosurgery*. 1988;22(4):694-702. doi:10.1227/00006123-198804000-00013. PMID:3259682
2. Couillard P, Karmi MZ, Abdelkader AM. Microsurgical removal of an intraventricular meningioma with ultrasound guidance, and balloon dilation of operative corridors: Case report and technical note. *Surg Neurol*. 1996;45(2):155-160. doi:10.1016/s0090-3019(96)80009-7. PMID:8607066
3. Schmid-Elsaesser R, Steiger HJ, Yousry T, Seelos KC, Reulen HJ. Radical resection of meningiomas and arteriovenous fistulas involving critical dural sinus segments: experience with in-traoperative sinus pressure monitoring and elective sinus reconstruction in 10 patients. *Neurosurgery*. 1997;41(5):1005-1016. doi:10.1097/00006123-199711000-00001. PMID:9361054
4. Black PM, Alexander E 3rd, Martin C, et al. Craniotomy for tumor treatment in an intraoperative magnetic resonance imaging unit. *Neurosurgery*. 1999;45(3):423-431. doi:10.1097/00006123-199909000-00001. PMID:10493363

5. Romstöck J, Strauss C, Fahlbusch R. Continuous electromyography monitoring of motor cranial nerves during cerebellopontine angle surgery. *J Neurosurg*. 2000;93(4):586-593. doi:10.3171/jns.2000.93.4.0586. PMID:11014536
6. Gorbach AM, Heiss JD, Kopylev L, Oldfield EH. Intraoperative infrared imaging of brain tumors. *J Neurosurg*. 2004;101(6):960-969. doi:10.3171/jns.2004.101.6.0960. PMID:15599965
7. Martorano P, Facco E, Falzetti G, Pelaia P. Spectral entropy assessment with auditory evoked potential in neuroanesthesia. *Clin Neurophysiol*. 2007;118(3):505-512. doi:10.1016/j.clinph.2006.11.002. PMID:17185033
8. Goto T, Muraoka H, Kodama K, et al. Intraoperative monitoring of motor evoked potential for the facial nerve using a cranial peg-screw electrode and a “threshold-level” stimulation method. *Skull Base*. 2010;20(6):429-434. doi:10.1055/s-0030-1261270. PMID:21772800
9. Kasper EM, Hess PE, Silasi M, et al. A pregnant female with a large intracranial mass: Reviewing the evidence to obtain management guidelines for intracranial meningiomas during pregnancy. *Surg Neurol Int*. 2010;1:95. doi:10.4103/2152-7806.74242. PMID:21245945
10. Ferroli P, Acerbi F, Albanese E, et al. Application of intraoperative indocyanine green angiography for CNS tumors: results on the first 100 cases. *Acta Neurochir Suppl*. 2011;109:251-257. doi:10.1007/978-3-211-99651-5_40. PMID:20960352
11. Li F, Lin J, Zhu G, et al. Neuroimaging and functional navigation as potential tools to reduce the incidence of surgical complications of lateral ventricular meningiomas. *Clin Neurol Neurosurg*. 2011;113(7):564-569. doi:10.1016/j.clineuro.2011.03.017. PMID:21530073
12. Matthies C, Raslan F, Schweitzer T, et al. Facial motor evoked potentials in cerebellopontine angle surgery: Technique, pitfalls and predictive value. *Clin Neurol Neurosurg*. 2011;113(10):872-879. doi:10.1016/j.clineuro.2011.06.011. PMID:21798660
13. Yang J, Liu YH, Ma SC, et al. Subtemporal transtentorial petrosalapex approach for giant petroclival meningiomas: analyzation and evaluation of the clinical application. *J Neurol Surg B Skull Base*. 2012;73(1):54-63. doi:10.1055/s-0032-1304557. PMID:23372996
14. Muroi C, Fandino J, Coluccia D, et al. 5-Aminolevulinic acid fluorescence-guided surgery for spinal meningioma. *World Neurosurg*. 2013;80(1-2):223.e1-3. doi:10.1016/j.wneu.2012.12.017. PMID:23247024
15. Della Puppa A, Rustemi O, Gioffrè G, et al. Application of indocyanine green video angiography in parasagittal meningioma surgery. *Neurosurg Focus*. 2014;36(2):E13. doi:10.3171/2013.12.FOCUS13385. PMID:24484251
16. Prada F, Del Bene M, Casali C, et al. Intraoperative navigated angiosonography for skull base tumor surgery. *World Neurosurg*. 2015;84(6):1699-1707. doi:10.1016/j.wneu.2015.07.025. PMID:26193670
17. Lekht I, Brauner N, Bakhsheshian J, et al. Versatile utilization of real-time intraoperative contrast-enhanced ultrasound in cranial neurosurgery: technical note and retrospective case series. *Neurosurg Focus*. 2016;40(3):E6. doi:10.3171/2015.11.FOCUS15570. PMID:26926064
18. Chakrabarti D, Surve RM, Bs D, Masapu D. Intraoperative aberrant bispectral index values due to facial nerve monitoring. *J Clin Anesth*. 2017;37:61-62. doi:10.1016/j.jclinane.2016.11.005. PMID:28235530
19. Acerbi F, Vetrano IG, Sattin T, et al. The role of indocyanine green videoangiography with FLOW 800 analysis for the surgical management of central nervous system tumors: an update. *Neurosurg Focus*. 2018;44(6):E6. doi:10.3171/2018.3.FOCUS1862. PMID:29852759

20. Güngör A, Danyeli AE, Akbaş A, et al. Ventricular Meningiomas: Surgical Strategies and a New Finding That Suggest an Origin From the Choroid Plexus Epithelium. *World Neurosurg*. 2019;129:e177-e190. doi:10.1016/j.wneu.2019.05.092. PMID:31121376
21. Bailo M, Gagliardi F, Boari N, et al. The role of surgery in meningiomas. *Curr Treat Options Neurol*. 2019;21(10):51. doi:10.1007/s11940-019-0587-9. PMID:31560106
22. Lepski G, Arévalo A, Roser F, et al. Electrophysiological predictors of hearing deterioration based on AEP monitoring during petroclival meningioma resection. *Neurosurg Rev*. 2021;44(3):1601-1609. doi:10.1007/s10143-020-01350-y. PMID:32671694
23. Ibn Essayed W, Aboud E, Al-Mefty O. Foramen Magnum Meningioma—The Attainment of the Intra-Arachnoidal Dissection: 2-Dimensional Operative Video. *Oper Neurosurg*. 2021;21(6):E518-E519. doi:10.1093/ons/opab317. PMID:34498699
24. D'Ercole M, D'Alessandris QG, Di Domenico M, et al. Is There a Role for Intraoperative Neuromonitoring in Intradural Extramedullary Spine Tumors? Results and Indications from an Institutional Series. *J Pers Med*. 2023;13(7):1103. doi:10.3390/jpm13071103. PMID:37511716
25. Silverstein JW, Shah HA, Ellis JA, D'Amico RS. Neuromonitoring Guided Vessel Identification in Iatrogenic Arterial Injury During Meningioma Resection. *Neurodiagn J*. 2023;63(1):47-57. doi:10.1080/21646821.2023.2168971
26. Silverstein JW, Duehr J, Vilaysom S, et al. Detecting and addressing secondary neural injuries in cranial surgery: Case report. *Neurodiagn J*. 2024. doi:10.1080/21646821.2024.2388954. PMID:39163123
27. Mori F, Sumi K, Watanabe M, et al. Intraoperative neuromonitoring of visual evoked potentials in a pregnant patient with meningioma: a case report. *BMC Neurol*. 2024;24(1):414. doi:10.1186/s12883-024-03915-6. PMID:39455945
28. Dadario NB, Tabor JK, Silverstein J, Sun XR, DAmico RS. Postoperative Focal Lower Extremity Supplementary Motor Area Syndrome: Case Report and Review of the Literature. Neurodiagn J. 2021 Dec;61(4):169-185. doi: 10.1080/21646821.2021.1991716. Epub 2021 Nov 15. PMID: 34781833.
